# Combining Heart Rate Variability with Disease Severity Score Variables for Mortality Risk Stratification in Septic Patients Presenting at the Emergency Department

**DOI:** 10.3390/ijerph16101725

**Published:** 2019-05-16

**Authors:** Jeremy Zhenwen Pong, Stephanie Fook-Chong, Zhi Xiong Koh, Mas’uud Ibnu Samsudin, Takashi Tagami, Calvin J. Chiew, Ting Hway Wong, Andrew Fu Wah Ho, Marcus Eng Hock Ong, Nan Liu

**Affiliations:** 1 Duke-NUS Medical School, National University of Singapore, Singapore 169857, Singapore; jeremypong@u.duke.nus.edu (J.Z.P.); marcus.ong.e.h@singhealth.com.sg (M.E.H.O.); 2 Health Services Research Unit, Singapore General Hospital, Singapore 169608, Singapore; stephanie.fook.m.c@sgh.com.sg; 3 Department of Emergency Medicine, Singapore General Hospital, Singapore 169608, Singapore; koh.zhixiong@singhealth.com.sg (Z.X.K.); andrew.ho@mohh.com.sg (A.F.W.H.); 4 General Medicine, Ministry of Health Holdings, Singapore 099253, Singapore; masuud.ibnusamsudin@mohh.com.sg; 5 Department of Emergency and Critical Care Medicine, Nippon Medical School Tama Nagayama Hospital, Tokyo 206-8512, Japan; t-tagami@nms.ac.jp; 6 Preventive Medicine Residency Program, National University Health System, Singapore 119228, Singapore; calvin.chiew@mohh.com.sg; 7 Department of General Surgery, Singapore General Hospital, Singapore 169608, Singapore; wong.ting.hway@singhealth.com.sg; 8 Health Services Research Centre, Singapore Health Services, Singapore 169856, Singapore

**Keywords:** heart rate variability, sepsis, mortality, prediction, emergency department

## Abstract

The emergency department (ED) serves as the first point of hospital contact for many septic patients, where risk-stratification would be invaluable. We devised a combination model incorporating demographic, clinical, and heart rate variability (HRV) parameters, alongside individual variables of the Sequential Organ Failure Assessment (SOFA), Acute Physiology and Chronic Health Evaluation II (APACHE II), and Mortality in Emergency Department Sepsis (MEDS) scores for mortality risk-stratification. ED patients fulfilling systemic inflammatory response syndrome criteria were recruited. National Early Warning Score (NEWS), Modified Early Warning Score (MEWS), quick SOFA (qSOFA), SOFA, APACHE II, and MEDS scores were calculated. For the prediction of 30-day in-hospital mortality, combination model performed with an area under the receiver operating characteristic curve of 0.91 (95% confidence interval (CI): 0.88–0.95), outperforming NEWS (0.70, 95% CI: 0.63–0.77), MEWS (0.61, 95% CI 0.53–0.69), qSOFA (0.70, 95% CI 0.63–0.77), SOFA (0.74, 95% CI: 0.67–0.80), APACHE II (0.76, 95% CI: 0.69–0.82), and MEDS scores (0.86, 95% CI: 0.81–0.90). The combination model had an optimal sensitivity and specificity of 91.4% (95% CI: 81.6–96.5%) and 77.9% (95% CI: 72.6–82.4%), respectively. A combination model incorporating clinical, HRV, and disease severity score variables showed superior predictive ability for the mortality risk-stratification of septic patients presenting at the ED.

## 1. Introduction

Sepsis is a life-threatening organ dysfunction that arises from a dysregulated host response to infection [1]. Worldwide, it has been estimated to impact up to 50 million people annually, potentially leading to five million deaths [2]. Even when it does not lead to mortality, sepsis can cause a myriad of complications that result in long-term morbidity [3], with significant implications for healthcare policy and resources. Although the diagnostic criteria of sepsis have been defined [1,4], sepsis severity and associated mortality risk exists in a spectrum, with no consensus on a method for grading or prognosticating it. As the emergency department (ED) is the first point of healthcare contact for many septic patients, an accurate tool for mortality risk-stratification would be invaluable in guiding management.

The ability to prognosticate and risk-stratify septic patients presenting at the ED is manifold. For the individual patient, risk-stratification can guide triage priority, the intended aggressiveness of management, candidacy for invasive monitoring or procedures, and disposition from the ED. In an administrative setting, accurate risk-stratification allows for prudent resource allocation and appropriate classification for research or administrative purposes. Indeed, objective estimates of mortality risk have been noted to provide greater certainty for the expected clinical course, allowing for management decisions to be made more confidently [5]. Scoring systems have also been shown to aid in the optimization and cost-effective use of healthcare resources [4,6].

Disease severity scoring systems have been developed to assess severity of illness and to predict the risk of mortality. Several of the more widely used scores include the National Early Warning Score (NEWS) [7], Modified Early Warning Score (MEWS) [8], Sequential Organ Failure Assessment (SOFA) [9], quick SOFA (qSOFA) [10], Acute Physiology and Chronic Health Evaluation II (APACHE II) [11], and the Mortality in Emergency Department Sepsis (MEDS) score [12]. The scores differ in their intended population of use (septic patients versus the general population), intended location of use (ED versus intensive care unit versus general use) and in how much information, and hence time, is required for their calculation. Studies that have utilized these scoring systems in the population of septic patients presenting at the ED have been promising, with the purpose-built MEDS score and the more elaborate scoring systems such as SOFA and APACHE II exhibiting superior predictive abilities [13,14,15,16]. 

Heart rate variability (HRV) analysis is a technique that utilizes electrocardiogram (ECG) tracings to examine beat-to-beat variations in heartbeats [17]. This provides a quick and non-invasive method of evaluating the autonomic modulation of the cardiovascular system, which has been shown to be dysregulated in sepsis [18]. Abnormalities in HRV parameters present themselves far before clinical signs become apparent [19,20] and have been found to correlate well with subsequent patient deterioration and mortality [13,21,22,23,24,25,26]. HRV analysis may thus represent a potentially useful method for the early risk-stratification of septic patients presenting at the ED and can be considered as a standalone predictor or as an augmentation of the other disease severity scores. 

In this study, we aimed to devise a model incorporating clinically relevant and best-performing demographic, clinical, and HRV parameters, along with the individual variables of SOFA, APACHE II, and MEDS scores for the prediction of 30-day in-hospital mortality (IHM) in septic patients presenting to the ED. We also aimed to create a model for rapid triage with variables that can be quickly ascertained on initial patient presentation.

## 2. Materials and Methods

### 2.1. Study Design and Setting

We conducted a retrospective data analysis on a convenience sample of patients presenting to the ED of Singapore General Hospital (SGH) between September 2014 to April 2017. The study was approved by SingHealth Centralized Institutional Review Board with a waiver of patient consent (CIRB Ref No.: 2016/2858). SGH is a tertiary care hospital in Singapore, with 300 to 500 patients seen in the ED per day. On presentation at the ED, patients are triaged with the national Singaporean Patient Acuity Category Scale (PACS), a symptom-based triage system without strict physiological criteria. Patients are assigned a PACS score ranging from 1 to 4, reflecting the urgency of their need for consultation with a physician. Patients triaged to PACS 1 are critically ill, those to PACS 2 are non-ambulant, those to PACS 3 are ambulant, and those to PACS 4 are non-emergencies. 

### 2.2. Study Population and Eligibility

Adult patients (aged 18 years and above) presenting to the ED, clinically suspected to have sepsis by their treating physicians and fulfilling at least two of the four systemic inflammatory response syndrome (SIRS) criteria [4] were recruited. The four SIRS criteria are a temperature of >38 °C or <36 °C, an elevated heart rate >90 beats per minute, a respiratory rate >20 breaths per minute, and a total white blood cell count >12,000 cells per mm^3^ or <4000 cells per mm^3^. Although a recent revision in 2016 was made to the criteria for sepsis with a recommendation to use qSOFA [1], the SIRS criteria of 1991 was used to define the septic population in this study. This is based on subsequent evidence that has found qSOFA to have poor sensitivity for septic patients, calling into question its suitability for ED sepsis screening as compared to the SIRS criteria [27,28,29,30]. Utilizing the SIRS criteria to define the septic population also allows for comparability with previously conducted studies. 

Patients who had ECG readings which were not suitable for HRV analysis were excluded. Inapplicable ECG readings included non-sinus rhythms (asystole, ventricular or supra-ventricular arrhythmias) or readings with a high proportion of artefacts or ectopics exceeding 30% of the recording. Patients lost to follow-up or transferred to other hospitals within 30 days of initial ED presentation were also excluded. The study was limited to patients triaged to PACS 1 or 2.

### 2.3. Data Collection 

Patients who met inclusion criteria received six-minute one-lead ECG monitoring immediately upon triage with the X-Series Monitor (ZOLL Medical Corporation, Chelmsford, MA, USA). ECG recordings were subsequently loaded onto the Kubios HRV program version 2.2 (Kuopio, Finland) for analysis. Noise removal was performed to suppresses high-frequency interference and low-frequency variations due to baseline wander and motion artefacts. RR interval sequences were generated for the computation of HRV parameters of the time domain, frequency domain and non-linear variables. Time-domain parameters quantify variability between successive heart beats, frequency-domain parameters quantify the signal energy in each of the three frequency bands (very low, low, and high frequency), while non-linear parameters quantify the organization and complexity of the time series [31]. A comprehensive description of HRV parameters can be found in the paper by Shaffer and colleagues [31]. 

Patient demographic data, comorbidities, clinical and laboratory parameters and treatment received for the duration of the patient’s ED stay were retrieved from the electronic medical records. The primary outcome was 30-day IHM following ED presentation.

### 2.4. Disease Severity Score Calculation

NEWS, MEWS and qSOFA were calculated with the first recorded value obtained upon patient presentation. SOFA, APACHE II and MEDS score utilized the most abnormal value recorded for the duration of the patient’s ED stay. For patients who did not have their NN triangular index (HRV parameter) calculated or serum bilirubin values obtained, the median value of the other patients served as a replacement. Parameters required for the calculation of NEWS, MEWS, qSOFA, SOFA, APACHE II, and MEDS score are listed in the Appendix A Table A1.

For the purposes of disease severity score calculations, we defined variables with reference to their original papers [7,8,9,10,11,12] unless stated otherwise. Both SOFA and APACHE II utilize the partial pressure of arterial oxygen (P_a_O_2_) as a component of their scores. As an arterial blood gas (ABG) is required for data on P_a_O_2_ and is infrequently performed, peripheral capillary oxygen saturation (S_p_O_2_) was used as a surrogate measurement where P_a_O_2_ was not available—a technique that has been described in the literature [21,32]. APACHE II incorporates pH as one of its variables but allows for the use of serum bicarbonate as a surrogate when pH data is unavailable [21]. In the clarification of several MEDS score variables, a patient was considered to have an altered mental state (AMS) if they had a Glasgow coma scale (GCS) score of less than 15, or if it was noted that they were ‘drowsy’ or ‘confused’ in their records. As our hospital does not routinely check for percentage bands on differential count, they were assumed to be normal if not reported. 

### 2.5. Statistical Analysis

Statistical analysis was conducted using SPSS version 25 (IBM corporation, Armonk, NY, USA). Univariable analyses of patient baseline characteristics, clinical parameters, and HRV measurements were reported by primary outcome (30-day IHM). Continuous variables were presented as mean (standard deviation) and compared between groups with the Mann–Whitney U test. Categorical variables were presented as a number (percentage) and compared between groups with Pearson’s chi-squared test or Fisher’s exact test as appropriate. Univariable analysis was similarly conducted for the individual components of the SOFA, APACHE II, and MEDS score, with their p-value guiding their inclusion or not into the multivariable logistic regression model.

Two logistic regression models were created for the prediction of the primary outcome. In the first model (rapid triage model), variables which could be ascertained within six minutes of patient presentation and with a *p*-value ≤0.2 for univariable analysis were considered as possible covariables for logistic regression. Such a model would have use for rapid triage while awaiting investigative data. The second model (combination model) considered demographic, clinical, and HRV parameters as well as the individual variables of the SOFA, MEDS score and APACHE II with a *p*-value ≤0.2 on univariable analysis and with non-zero counts as possible covariables for logistic regression. Clinical judgement was utilized to deconflict similar variables, and continuous variables were chosen over categorical variables where possible. In the combination model, we utilize the entirely of information garnered during a patient’s ED stay to maximize the predictive ability for the primary outcome.

The predictive performance of disease severity scores was assessed alongside logistic regression models for receiver operating characteristic (ROC) analysis. Sensitivities, specificities, positive and negative predictive values for each score were determined at the optimal cut-off point as indicated by the point on the ROC curve nearest to the upper left corner of the ROC graph. 

## 3. Results

### 3.1. Patient Recruitment and Outcomes

**Figure 1:**
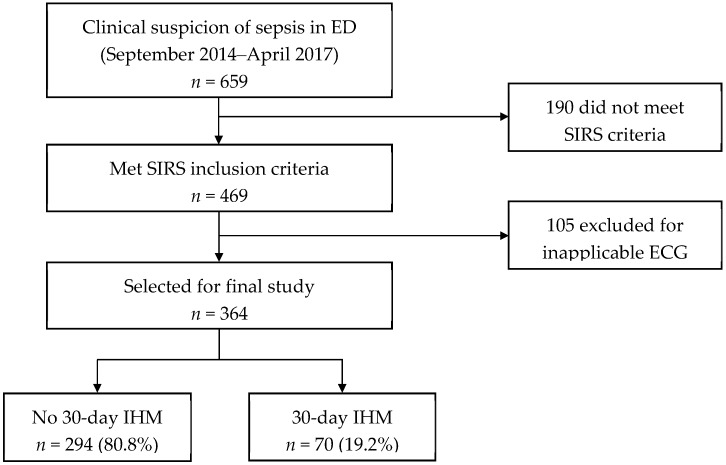
Patient recruitment flowchart. ECG: electrocardiogram; ED: emergency department; IHM: in-hospital mortality; SIRS: systemic inflammatory response syndrome.

Figure 1 shows the patient recruitment flow chart. A total of 659 patients clinically suspected to have sepsis presented at the ED between September 2014 to April 2017. One hundred and ninety patients did not meet the criteria for SIRS and were excluded. Of the 469 patients that met SIRS criteria, 105 were excluded for having an inapplicable ECG reading. Three hundred and sixty-four patients were selected for the final study, with 70 (19.2%) meeting the primary outcome of 30-day IHM.

### 3.2. Baseline Characteristics, Clinical and Heart Rate Variability Parameters

Univariable analysis of patient baseline characteristics and clinical parameters are presented in Table 1. Patients who met the primary outcome were older and presented with a lower systolic blood pressure, higher respiratory rate, lower temperature and lower GCS. During their stay in the ED, the worst recorded values of systolic blood pressure, respiratory rate and GCS were also more abnormal for this group of patients. 

Univariable analyses of HRV parameters are shown in Table 2. Patients who met the primary outcome had an increased standard deviation of NN (beat-to-beat) time intervals (SD NN), standard deviation of heart rate (SD HR), root mean square of successive NN interval differences (RMSSD), number of pairs of successive NN intervals that differ by more than 50 milliseconds (NN50), proportion of successive NN intervals that differ by more than 50 milliseconds (pNN50), baseline width of the NN interval histogram (TINN), high frequency (HF) power (ms^2^), HF power (nu) and Poincaré plot standard deviation (SD) 1. Parameters which were decreased include low-frequency (LF) power (nu), LF/HF, detrended fluctuation analysis (DFA) alpha 1 and DFA alpha 2. 

### 3.3. Components of Logistic Regression Models 

Table 3 shows independent predictors of 30-day IHM as included in the combination model and the rapid triage model. The combination model is comprised of two clinical variables (presenting temperature and the worst respiratory rate recorded), one HRV parameter (Poincaré plot standard deviation 2), four MEDS score variables (terminal illness, lower respiratory tract infection (LRTI) suspicion, respiratory distress, and septic shock), and one SOFA variable (platelet count). The rapid triage model is comprised of four clinical variables (presenting systolic blood pressure, respiratory rate, temperature, and GCS) and one HRV parameter (DFA alpha 2). Collinearity testing revealed no multicollinearity between independent predictors for both models (data not shown).

### 3.4. Prediction of Primary Outcome

**Figure 2:**
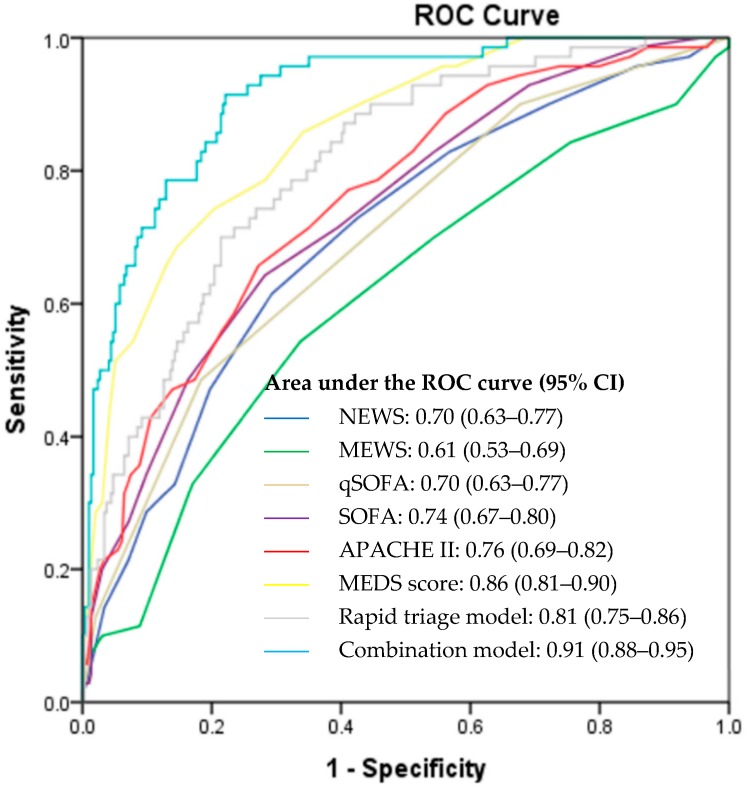
Receiver operator characteristic (ROC) curves for the prediction of 30-day IHM. APACHE II: Acute Physiology and Chronic Health Evaluation II; CI: confidence interval; IHM: in-hospital mortality; MEDS: Mortality in Emergency Department Sepsis; MEWS: Modified Early Warning Score; NEWS: National Early Warning Score; qSOFA: Quick Sequential Organ Failure Assessment; ROC: receiver operating characteristic; SOFA: Sequential Organ Failure Assessment.

Figure 2 illustrates the ROC curves of the rapid triage model, combination model and calculated disease severity scores for the prediction of 30-day IHM. Among the scores which utilized information determined from the initial patient presentation, the rapid triage model outperformed NEWS, MEWS, and qSOFA with an area under the ROC curve (AUC) of 0.81 (95% CI: 0.75–0.86), 0.70 (95% CI: 0.63–0.77), 0.61 (95% CI: 0.53–0.69), and 0.70 (95% CI: 0.63–0.77), respectively. For scoring systems which utilized information obtained throughout the patient’s ED stay, the combination model displayed superior predictive ability as compared to SOFA, APACHE II, and MEDS scores, with an AUC of 0.91 (95% CI: 0.88–0.95), 0.74 (95% CI: 0.67–0.80), 0.76 (95% CI: 0.69–0.82), and 0.86 (95% CI: 0.81–0.90), respectively.

Table 4 displays the sensitivities, specificities, positive predictive values (PPV), and negative predictive values (NPV) of the rapid triage model, combination model, and calculated disease severity scores. The rapid triage model sensitivity, specificity, PPV and NPV were 68.6%, 78.6%, 43.2%, and 91.3%, respectively, while the combination model sensitivity, specificity, PPV and NPV were 91.4%, 77.9%, 49.6%, and 97.4%, respectively.

## 4. Discussion

In this study, we derived a combination model which exhibits superior predictive ability over established disease severity scoring systems for the prediction of 30-day IHM in septic patients presenting at the ED. A second model (rapid triage model) that can be quickly determined on patient presentation outperforms commonly used quick scoring systems (qSOFA, NEWS, and MEWS) and even the comprehensive scores (SOFA and APACHE II) while requiring less time, information and procedures to derive. With their enhanced predictive ability and ease of use, the models created can be used to risk-stratify the patient at two crucial timepoints: early risk-stratification with the rapid triage model on presentation, and re-stratification with the combination model for improved accuracy on transfer out of the ED.

In the creation of the combination model, we sought to maximize the predictive ability in a scoring system which utilized all available information gathered over a patient’s time at the ED. The combination model achieved an AUC of 0.91 (95% CI: 0.88–0.95) and included two clinical variables (presenting temperature and worst respiratory rate recorded), one HRV parameter (Poincaré plot standard deviation 2), four MEDS score variables (terminal illness, LRTI suspicion, respiratory distress, and septic shock) and one SOFA variable (platelet count). The predictive performance of the combination model exceeded the other disease severity scores, and the variables included can be used as information regarding significant factors which influence mortality. MEDS score variables included in the combination score had the strongest predictive values (respiratory distress adjusted odds ratio (aOR): 7.17 (95% CI: 1.88–27.40); terminal illness aOR: 5.93 (95% CI: 2.85–12.35)) while the HRV parameter included contributed to a lesser extent (Poincaré plot standard deviation 2 aOR: 1.03 (95% CI: 1.01–1.04)). The creation of the combination model, however, may have been limited by the study sample size. APACHE II and SOFA variables were ordinal and included up to eight ordinal categories in which patients would be sorted. As we excluded variables that had zero counts for any category, several APACHE II variables (mean arterial pressure, respiratory distress, and serum sodium) were not considered as covariables in the creation of the combination model even when found to be statistically different on univariable analysis. The predictive ability of the combination model may therefore be further enhanced given a larger sample size that would allow for the consideration of the omitted variables. 

The rapid triage model utilizes parameters which can be readily ascertained on initial patient presentation. Demographic characteristics and vital signs have long been used for a quick assessment of the patient but can be supplemented with HRV analysis. On logistic regression, HRV parameter DFA alpha 2 was included together with presenting systolic blood pressure, respiratory rate, temperature, and GCS as independent predictors of 30-day IHM. Among the independent predictors, DFA alpha 2 had the strongest predictive value (aOR: 0.28 (95% CI: 0.14–0.57)), emphasizing the utility of HRV analysis in supplementing information derived from the traditional vital signs. DFA describes the relationship between successive RR intervals over different time frames, with DFA alpha 1 representing short-term fluctuations and DFA alpha 2 representing long-term fluctuations [31]. Although the physiological meaning of DFA has not been well elucidated, it has been postulated that short-term fluctuations represent the baroreceptor reflex, while long-term fluctuations reflect regulatory mechanisms of the heart beat cycle [31]. DFA has also been found to be related to LF power, HF power, and the LF/HF ratio [33], possibly integrating the interplay of sympathetic and parasympathetic influences into its calculations. In our study, an increase in DFA alpha 2 is associated with a significant decrease in the odds for 30-day IHM. The ability of DFA to predict deterioration or risk of mortality in septic patients is consistent with other studies [20,25]. Apart from its use in septic patients, DFA has also been found to be a predictor for outcome after myocardial infarction [34] and in heart failure [35].

The performance of the established disease severity scores in our patient population may also be assessed in this study. We note that scores which utilized basic, easily obtainable parameters (NEWS, MEWS, and qSOFA) had poorer performance on ROC analysis as compared to scores which included more detailed information (SOFA, APACHE II, and MEDS score). The more comprehensive scores offered better predictive capability, at the cost of additional time and procedures required for the gathering of information. Among the rapid scoring systems, qSOFA (AUC: 0.70) and NEWS (AUC: 0.70) exhibited an improved predictive ability as compared to MEWS (AUC: 0.61). Among the comprehensive scoring systems, MEDS score (AUC: 0.86) exhibited a superior predictive ability as compared to SOFA (AUC: 0.74) and APACHE II (AUC: 0.76). It is interesting to note that qSOFA, which is comprised of only three variables (GCS < 15, respiratory rate ≥ 22, and systolic blood pressure ≤ 100) to be selected in binary format (yes or no), shows an appreciable predictive ability for 30-day IHM and has an AUC superior to NEWS and similar to MEWS, both of which require more variables for calculation. The utility and applicability of qSOFA for initial triage, which can be employed quickly and with minimal information, is thus highlighted in this study. Among the comprehensive scoring systems, the MEDS score demonstrated the highest AUC. This is not unexpected as the MEDS score was purpose-built for use in the population of septic patients presenting at the ED. SOFA and APACHE II were developed for use in the general ICU population, and although both perform well, they require more invasive procedures for their calculations.

There are several limitations to this study. First, our predictive models were able to achieve good AUC values on derivation but will require external validations to evaluate their effectiveness. Second, we excluded a sizable portion of septic patients (22.4%) due to ECG readings which were not suitable for HRV analysis. This patient group would likely have a distinct clinical profile, and our interpretation of findings are therefore limited only to patients with applicable ECG readings. Third, as our study was limited to sicker patients triaged to PACS 1 and 2, further studies will be required for generalization to all patients presenting at the ED. Our future work aims to enhance the model’s robustness by utilizing novel heart rate n-variability (HRnV) measures [36] to better interrogate the autonomic modulation of the cardiovascular system during sepsis. These additional HRnV measurements could improve the predictive ability of conventional HRV-based models.

## 5. Conclusions

A combination model incorporating best-performing clinical and HRV parameters alongside individual components of the SOFA and MEDS score shows a superior predictive ability for the mortality risk-stratification (30-day IHM) of septic patients presenting at the ED. With its enhanced predictive ability and practical ease of use, the combination model can provide additional information on the severity of sepsis and be considered for implementation to inform diagnostic and therapeutic decisions, patient disposition and resource allocation, as well as for patient classification for administrative or research purposes.

## Figures and Tables

**Table 1:** Baseline characteristics and clinical parameters.

Variables	No 30-Day IHM (*n* = 294)	30-Day IHM (*n* = 70)	*p*-Value
Age in years, mean (SD)	65.7 (16.4)	72.8 (15.0)	0.001
Gender, *n* (%)			0.595
Male	147 (50.0)	32 (45.7)	
Female	147 (50.0)	38 (54.3)	
Race, *n* (%)			0.659
Chinese	213 (72.4)	53 (75.7)	
Malay	44 (15.0)	7 (10.0)	
Indian	22 (7.5)	7 (10.0)	
Other	15 (5.1)	3 (4.3)	
Medical history, *n* (%)			
Ischemic heart disease	76 (25.9)	23 (32.9)	0.236
Diabetes	118 (40.1)	27 (38.6)	0.892
Hypertension	162 (55.1)	35 (50.0)	0.505
Cancer	88 (29.9)	25 (35.7)	0.389
Serious infection	121 (41.2)	30 (42.9)	0.789
Drug history, *n* (%)			
Beta-blocker	96 (32.7)	16 (22.9)	0.116
Digoxin	11 (3.7)	2 (2.9)	1.000
Calcium channel blocker	73 (24.8)	17 (24.3)	1.000
Amiodarone	4 (1.4)	0 (0.0)	1.000
Vital signs at presentation, mean (SD)			
Heart rate, bpm	117.5 (21.8)	116.7 (23.4)	0.622
Systolic blood pressure, mm Hg	115.5 (33.1)	103.3 (31.1)	0.004
Diastolic blood pressure, mm Hg	63.4 (17.7)	60.2 (16.0)	0.200
Respiratory rate, bpm	20.2 (3.9)	23.4 (5.7)	<0.001
Temperature, °C	38.1 (1.2)	37.2 (1.4)	<0.001
Glasgow coma scale (3–15)	13.4 (3.0)	12.2 (3.9)	0.002
Vital signs worst recorded, mean (SD)			
Systolic blood pressure, mm Hg	96.0 (25.3)	80.5 (21.3)	<0.001
Respiratory rate, bpm	22.8 (5.3)	27.9 (6.8)	<0.001
Glasgow coma scale (3–15)	13.2 (3.3)	12.1 (3.9)	0.002
WBC count, mean (SD)	14.3 (7.2)	14.0 (10.2)	0.291
Source of infection, *n* (%)			0.041
Respiratory	83 (28.2)	32 (45.7)	
Urinary tract	71 (24.1)	6 (8.6)	
Gastrointestinal	16 (5.4)	5 (7.1)	
Musculoskeletal	11 (3.7)	2 (2.9)	
Hepatobiliary	21 (7.1)	3 (4.3)	
Peritoneum	3 (1.0)	1 (1.4)	
Skin	10 (3.4)	0 (0.0)	
Line	9 (3.1)	0 (0.0)	
Cardiac	6 (2.0)	2 (2.9)	
Central nervous system	1 (0.3)	0 (0.0)	
Unknown	45 (15.3)	16 (22.9)	
No infection	18 (6.1)	3 (4.3)	
Disposition from ED, *n* (%)			<0.001
General ward	251 (85.4)	52 (74.3)	
High dependency	26 (8.8)	3 (4.3)	
Intensive care unit	17 (5.8)	15 (21.4)	

ED: emergency department; IHM: in-hospital mortality; WBC: white blood cell.

**Table 2:** Heart rate variability parameters.

Variable, Mean (SD)	No 30-Day IHM (*n* = 294)	30-Day IHM (*n* = 70)	*p*-Value
**Time domain**			
Mean NN, s	559.20 (117.90)	561.47 (130.81)	0.866
Standard deviation of NN, s	21.96 (24.85)	31.98 (34.38)	0.050
Mean heart rate, bpm	111.95 (21.44)	112.79 (23.93)	0.742
Standard deviation of heart rate, bpm	4.84 (5.87)	6.78 (7.37)	0.011
RMSSD, s	26.56 (39.08)	42.66 (50.00)	<0.001
NN50, n	48.60 (114.22)	72.80 (121.94)	<0.001
pNN50, %	7.47 (17.43)	11.98 (20.12)	<0.001
NN triangular index	3.87 (3.45)	4.55 (5.39)	0.918
TINN	137.60 (147.02)	194.86 (180.16)	0.004
**Frequency domain**			
Total power, ms^2^	514.81 (1737.84)	1276.95 (3254.90)	0.176
VLF power, ms^2^	117.57 (307.02)	254.08 (833.86)	0.939
LF power, ms^2^	120.47 (476.99)	305.57 (826.58)	0.485
HF power, ms^2^	274.43 (1005.67)	713.44 (1763.77)	0.011
LF power, nu	47.32 (28.82)	35.46 (25.76)	0.002
HF power, nu	51.93 (28.33)	63.73 (25.44)	0.002
LF/HF	2.59 (4.47)	1.65 (4.88)	0.002
**Non-linear domain**			
Poincaré plot standard deviation 1, ms	18.80 (27.66)	30.19 (35.39)	<0.001
Poincaré plot standard deviation 2, ms	23.05 (23.39)	32.42 (34.39)	0.147
Approximate entropy	0.98 (0.34)	1.02 (0.35)	0.304
Sample entropy	1.07 (0.55)	1.13 (0.59)	0.454
DFA, alpha 1	0.67 (0.38)	0.54 (0.27)	0.016
DFA, alpha 2	0.95 (0.42)	0.71 (0.41)	<0.001

DFA: detrended fluctuation analysis; HF: high frequency; IHM: in-hospital mortality; LF: low frequency; NN: beat-to-beat time interval; NN50: number of pairs of successive NN intervals that differ by more than 50 milliseconds; pNN50: proportion of successive NN intervals that differ by more than 50 milliseconds; RMSSD: root mean square of successive NN interval differences; TINN: baseline width of the NN interval histogram; VLF: very low frequency.

**Table 3:** Multivariable logistic regression models for prediction of 30-day IHM. The combination model utilizes information made available over the entire course of a patient’s emergency department stay. The rapid triage model utilizes variables that can be obtained within six minutes of patient presentation.

Variables	Combination Model	Rapid Triage Model
	Adjusted Odds Ratio (95% CI)	
**Clinical parameters**		
Systolic blood pressure (presenting)		0.99 (0.98–1.00)
Respiratory rate (presenting)		1.13 (1.06–1.20)
Temperature (presenting)	0.61 (0.45–0.81)	0.60 (0.47–0.77)
Glasgow coma scale (presenting)		0.91 (0.84–0.99)
Respiratory rate (worst)	1.09 (1.02–1.16)	
**HRV parameters**		
Poincaré plot standard deviation 2	1.03 (1.01–1.04)	
DFA, alpha 2		0.28 (0.14–0.57)
**MEDS score variables**		
Terminal illness	5.93 (2.85–12.35)	
LRTI suspicion	3.09 (1.45–6.59)	
Respiratory distress	7.17 (1.88–27.40)	
Septic shock	3.11 (1.51–6.37)	
**SOFA variables**		
Coagulation (platelet count)		
≥150,000	Reference	
100,00–149,999	0.54 (0.11–2.58)	
50,000–99,999	1.76 (0.48–6.52)	
20,000–49,999	9.43 (1.89–47.07)	
<20,000	1.19 (0.10–15.00)	

CI: confidence interval; DFA: detrended fluctuation analysis; HRV: heart rate variability; LRTI: lower respiratory tract infection; MEDS: mortality in emergency department sepsis; SOFA: sequential organ failure assessment.

**Table 4:** Performance of scoring systems for the prediction of 30-day IHM.

Clinical Scores	Sensitivity, % (95% CI)	Specificity, % (95% CI)	PPV, % (95% CI)	NPV, % (95% CI)
**Quick scoring systems**				
Rapid triage model ≥ 0.201	68.6 (56.2–78.9)	78.6 (73.3–83.0)	43.2 (34.0–53.0)	91.3 (87.0–94.3)
NEWS ≥ 8	61.4 (49.0–72.8)	70.8 (65.2–75.9)	33.3 (27.9–39.3)	88.5 (85.0–91.3)
MEWS ≥ 6	54.3 (41.9–66.3)	66.3 (60.6–71.7)	27.7 (22.7–33.4)	85.9 (82.3–88.9)
qSOFA ≥ 2	48.6 (36.4–60.8)	81.6 (76.7–85.9)	38.6 (30.9–47.0)	87.0 (84.1–89.4)
**Comprehensive scoring systems**				
Combination model ≥ 0.156	91.4 (81.6–96.5)	77.9 (72.6–82.4)	49.6 (40.7–58.5)	97.4 (94.3–99.0)
APACHE II ≥ 23	65.7 (53.3–76.4)	72.8 (67.3–77.7)	36.5 (28.3–45.6)	89.9 (85.2–93.3)
SOFA ≥ 6	64.3 (51.9–75.4)	71.8 (66.3–76.8)	35.2 (29.6–41.1)	89.4 (85.9–92.1)
MEDS ≥ 12	74.3 (62.4–84.0)	79.6 (74.5–84.1)	46.4 (40.0–53.0)	92.9 (89.7–95.1)

NPV: negative predictive value; PPV: positive predictive value.

## Appendix A

**Table A1:** Information required for the calculation of disease severity scores.

Variables	NEWS	MEWS	qSOFA	SOFA	APACHE II	MEDS Score
Age					✓	✓
Nursing home resident						✓
Chronic health problems					✓	
Terminal illness						✓
Heart rate	✓	✓			✓	
Blood pressure	✓	✓	✓	✓	✓	✓
Respiratory rate/supplementary oxygen	✓	✓	✓		✓	✓
Temperature	✓	✓			✓	
P_a_O_2_				✓	✓	
S_p_O_2_	✓					✓
F_i_O_2_				✓	✓	
AVPU	✓	✓				
GCS			✓	✓	✓	
AMS						✓
Hematocrit					✓	
White blood cell count					✓	
Percentage bands						✓
Platelet count				✓		✓
Serum sodium					✓	
Serum potassium					✓	
Serum creatinine				✓	✓	
Arterial pH or serum bicarbonate					✓	
Serum bilirubin				✓		
Suspicion of LRTI						✓
Septic shock						✓
Acute renal failure					✓	
Use of vasopressors				✓		

AMS: altered mental status; APACHE II: Acute Physiology and Chronic Health Evaluation II; AVPU: alert, verbal, pain, unresponsive; F_i_O_2_: fraction of inspired oxygen; GCS: Glasgow coma scale; LRTI: lower respiratory tract infection; MEDS: Mortality in Emergency Department Sepsis; MEWS: Modified Early Warning Score; NEWS: National Early Warning Score; P_a_O_2_: partial pressure of arterial oxygen; qSOFA: Quick Sequential Organ Failure Assessment; SOFA: Sequential Organ Failure Assessment; S_p_O_2_: peripheral capillary oxygen saturation.
